# Effective Low-Power Wearable Wireless Surface EMG Sensor Design Based on Analog-Compressed Sensing

**DOI:** 10.3390/s141224305

**Published:** 2014-12-17

**Authors:** Mohammadreza Balouchestani, Sridhar Krishnan

**Affiliations:** Electrical and Computer Engineering Department, Ryerson University, 350 Victoria Street, Toronto, ON M5B2K3, Canada; E-Mail: krishnan@ryerson.ca

**Keywords:** sEMG bio-signal, compressed sensing, random sensing dictionary, reconstruction process, sparsity

## Abstract

Surface Electromyography (sEMG) is a non-invasive measurement process that does not involve tools and instruments to break the skin or physically enter the body to investigate and evaluate the muscular activities produced by skeletal muscles. The main drawbacks of existing sEMG systems are: (1) they are not able to provide real-time monitoring; (2) they suffer from long processing time and low speed; (3) they are not effective for wireless healthcare systems because they consume huge power. In this work, we present an analog-based Compressed Sensing (CS) architecture, which consists of three novel algorithms for design and implementation of wearable wireless sEMG bio-sensor. At the transmitter side, two new algorithms are presented in order to apply the analog-CS theory before Analog to Digital Converter (ADC). At the receiver side, a robust reconstruction algorithm based on a combination of ℓ_1_-ℓ_1_-optimization and Block Sparse Bayesian Learning (BSBL) framework is presented to reconstruct the original bio-signals from the compressed bio-signals. The proposed architecture allows reducing the sampling rate to 25% of Nyquist Rate (NR). In addition, the proposed architecture reduces the power consumption to 40%, Percentage Residual Difference (PRD) to 24%, Root Mean Squared Error (RMSE) to 2%, and the computation time from 22 s to 9.01 s, which provide good background for establishing wearable wireless healthcare systems. The proposed architecture achieves robust performance in low Signal-to-Noise Ratio (SNR) for the reconstruction process.

## 1. Introduction

Today's healthcare systems are effective for an individual measurement from the human body, but are not integrated into comprehensive Body Area Networks (BANs), wherein simultaneous biomedical sensors work on a test subject. Furthermore, there is a need for increasing patient mobility because in many cases biomedical sensors for medical monitoring are not wireless yet [1]. This creates the need for the implementation of new wireless healthcare systems with a common architecture and the capacity to handle multiple wearable wireless bio-sensors, monitoring different body signals, with different requirements. Using wireless healthcare systems based on low-power wearable wireless bio-sensors has two aspects [1]. Firstly the use of new wireless technological solutions enables individually based, multi-parameter monitoring at home. Thus patients with chronic diseases, as well as a constantly growing number of seniors, will profit from treatment and medical monitoring at home or workplace. Moreover, unlimited mobility implies the use of wireless and even implanted bio-sensors, which greatly enhance home monitoring and follow-up of medical conditions. The second aspect emphasizes with increasing the efficiency of treatments at hospital and medical centers. The rising healthcare costs also make it an urgent need to develop ambulatory systems to reduce hospital visits. Therefore, it is necessary to design portable, low power, low sampling rate, and high-performance wireless health care systems that deliver healthcare services at any location 24/7. The sEMG method is a non-invasive and convenient procedure to record signal and machine-based estimation of force of muscle contraction or for assessing muscle fatigue with large number of rehabilitation and other applications such as myoelectric control, sensors placement, fatigue detection, and voice recognition [2]. Existing sEMG systems are based on wired/fixed sensors, and they suffer of long processing time, low speed, limited mobility, high cost, and large size [3,4]. These limits create real need for the implementation of new wearable wireless sEMG sensors that deliver healthcare services anywhere at any time [5].

The sEMG bio-signals can be processed to detect medical abnormalities or to analyze the biomechanics of the human or animal movement [6,7]. There are three important features in the sEMG bio-signal, *i.e.*, amplitude (ranging from 1 *μ*V to 50 mV), frequency, and phase. The sEMG signals exhibit good level of sparsity in the time and frequency domains [7]. The existing and conventional data acquisition approaches have traditionally relied on the Shannon sampling theorem [7]. This theory says a signal must be sampled at least twice its bandwidth in order to be represented without error. The traditional approaches have two major drawbacks. Firstly, they generate huge intolerable number of samples for many applications with a large bandwidth. Secondly, even for low signal bandwidths, including some biomedical signals, they produce a large amount of redundant digital samples. That is why it is desirable to reduce the number of acquired samples by utilizing sparsity. Compressive sampling (CS) is a random data acquisition procedure that requires only a few incoherent random linear measurements to compress signals that are sparse in some domain [7]. The CS theory is an optimal solution for this purpose. Specifically, the application of analog-CS before the ADC achieves compression of the data with a proportionate savings in energy. After an exhaustive search, we did not find any new study that aims at an effective random sampling rate data acquisition algorithm for wireless sEMG systems. This paper aims to apply analog-CS theory to establish low power and low sampling rate algorithms for a wearable wireless sEMG sensor for long-term recording of sEMG signals, which is a very useful tool for detection of various pathologies. This work is novel in four aspects: (1) compressing sEMG bio-signal at the sensing step before ADC; (2) digitalizing only compressed sEMG bio-signal; (3) transmitting the sEMG bio-data only when a significant event is detected; (4) reducing the number of bits transmitted to minimize the average radio power while maintaining the captured signal information. In this paper, we firstly apply the analog-CS theory as a new and random data acquisition procedure to the transmitter side before ADC in order to compress analog input signal. Secondly, we apply the Sensing Matrix Selection (SMS) method at the transmitter side in order to determine the best fit for random sensing matrix in the CS scenario. Thirdly, the reconstruction algorithm based on the contribution of ℓ_1_-ℓ_1_-optimization and BSBL framework is applied to the receiver side in order to recover the original sEMG bio-signals. The proposed architecture allows reducing the sampling rate to 25% of NR. In addition, the proposed architecture reduces the power consumption to 40%, PRD to 24%, RMSE to 2%, and the computation time from 22 s to 9.01 s, which provide good backgrounds for providing low sampling rate wireless healthcare services. Furthermore, the proposed architecture achieves robust performance in low SNR for the reconstruction algorithm. The proposed algorithms are created by C, HSPICE, and MATLAB/Simulink toolboxes. The proposed algorithms are tested over several hours of clinical sEMG bio-signals from the following databases: (1) PhysioNet (PhysioBank ATM) [8]; (2) EMG Bank [9]; (3) EMG project lab [10]. The structure of this paper is organized as follows: In Section II, an overview about CS theory as a random data acquisition method is presented. In Section III, the proposed system architecture and our novel algorithms for the transmitter and receiver sides of wireless sEMG sensor are presented. In Section IV, the results on sampling rate, power consumption, computation time, and reconstruction process are illustrated. The conclusion is given in Section V and future works are discussed in Section VI.

## 2. Overview of Compressed Sensing Theory

The need for compression of sEMG bio-signals arises in the following areas: (1) large sEMG database in the hospital; (2) long-term sEMG recording; (3) ambulatory (24 h) monitoring of sEMG bio-signals; (4) limits on hardware memory and delay; (5) establishing ultra low power and low sampling rate wireless healthcare sEMG systems [11–13].

The existing compression techniques rely on the Shannon sampling theorem and have two major drawbacks. Firstly, they generate large number of samples for many applications with a large bandwidth. Secondly, even for the bio-signals with low bandwidth, they produce a large amount of redundant digital samples. Table 1 compares the existing and random data acquisition methods based on the CS theory in terms of sampling frequency (*f_s_*), sampling rate, Compression Ratio (CR), and distortion degree [14]. Highlighted in Table 1 are the lower values for sampling rate and higher values for CR obtained by the analog-CS theory. The CS theory is a mathematical framework in acquiring and recovering for sparse signals with the help of an incoherent projecting basis that provides insight into how a high resolution dataset can be inferred from a relatively small and random number of measurements using simple random linear process. The basic idea of the CS theory as a new and random sampling approach is that when the biomedical signal is sparse or nearly sparse in terms of the number of non-zero coefficients, relatively few well-chosen observations suffice to reconstruct the original signal. In fact, CS is a new approach for the acquisition and recovery of sparse or compressive biomedical signals significantly below the classical Nyquist rate. Any signal can be expressed as [15]:

(1) $$D=\sum_{i=1}^{N}\left(S_{i}\Psi_{i}\right)$$

where *S* is the coefficient vector for *D* under the basis Ψ = [Ψ_1_, Ψ_2_, …, Ψ*_N_*]. If *D* has the most compact representation in Ψ, then *D* can be compressed in the proper basis [16]. The CS theory also proposes that rather than acquiring the entire signal and then performing compression, it should be possible to capture only a part of the signal's useful information. In the CS theory, we have a *M* x *N* measurement matrix Φ and biomedical signal *D* with *N* x 1 dimension such that *M* ≪ *N*; therefore, the compressed signal *C* can be expressed as [17]:

(2) $${\left[C\right]}_{M\times 1}={\left[\Phi \right]}_{M\times N}{\left[D\right]}_{N\times 1}$$

The original signals can be exactly reconstructed, with a high level of accuracy and probability via ℓ_1_ norm by solving the following convex optimization problem [18]:

(3) $$\text{min}{\left\|D\right\|}_{1}\;s.t\;C=\Phi D$$

where ‖*D*‖_1_ is defined as ‖ *D* ‖_1_ = Σ*_n_* ‖ *D_n_* ‖ with *D* ∊ ℜ*^N^*. However, certain conditions must be met to guarantee the accuracy of the reconstruction process [19,20].

### 2.1. Digital and Analog-CS Scenarios

**Figure 1.**
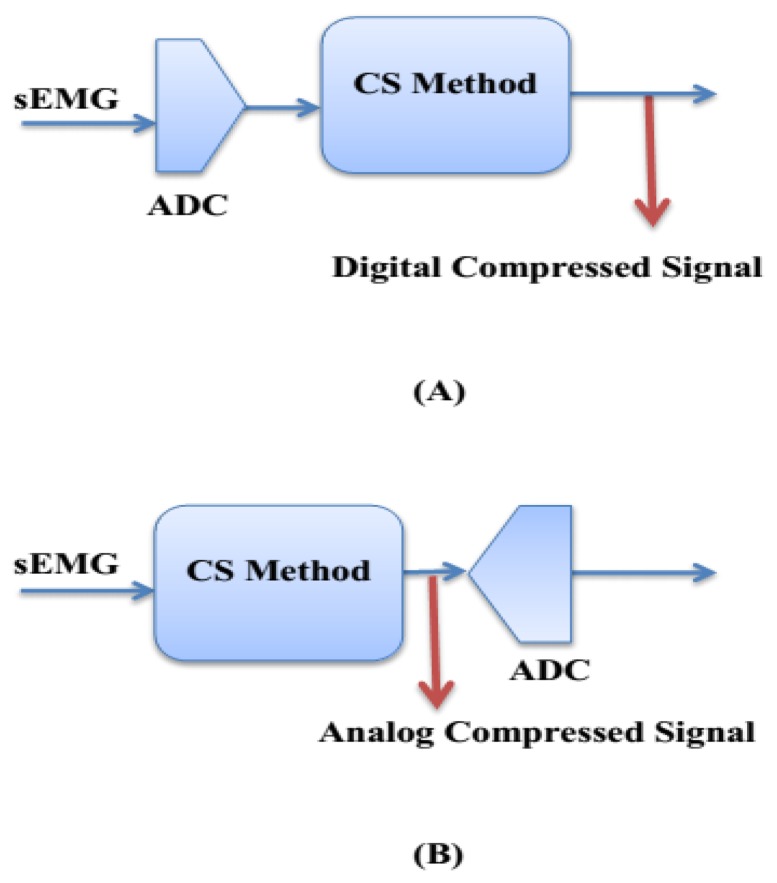
CS scenarios: (**A**) Digital (**B**) Analog.

The applications of wireless and wearable healthcare systems are constrained by the available energy, which imposes strict energy requirements on the bio-sensor circuits. That is why the following items are important to minimize power consumption: (1) compress bio-signal at the sensing step; (2) digitize only compressed bio-signal; (3) transmit data only when a significant event is detected; (4) reduce the number of bits transmitted to minimize the average radio power while maintaining the captured bio-signal information. Most of the power (78%) of the total power in an sEMG bio-sensor is dissipated in the transmitter side [21]. Therefore, there is a real need to decrease the amount of data to be transmitted and reduce the duty cycle of the transmitter to establish ultralow power bio-sensors. The analog-CS theory is an optimal solution that achieves significant improvements in minimizing sampling load as well as power consumption. Figure 1 shows the difference between analog-CS and digital-CS scenarios.

It can be seen in Figure 1 that the analog-CS scenario is based on Random demodulation (RD) using Gilbert multiplier [22]. This scenario consists of the following steps. (1) The analog bio-signal multiplies with the random sensing matrix *ϕ* based on Gilbert approach [22,23]; (2) The output signal of multiplier goes to an active integrator; (3) The *M* × 1 dimension compressed version of the input bio-signal is generated at the transmitter side. Therefore, in the analog-CS scenario at the transmitter side, the input analog sEMG bio-signal [*D*] _*N* × 1_ is compressed to output analog bio-signal [*C*] _*M* × 1_ such that *M* ≪ *N* and then, the ADC needs to digitize a smaller amount of data [24,25]. In the wireless sEMG systems, real-time sEMG bio-data is transmitted to a personal base station (e.g., a smart-phone, personal computer, or any portable device) and then transmitted to hospital, medical centers, and healthcare provider via the Internet.

## 3. The Proposed System Architecture

**Figure 2.**
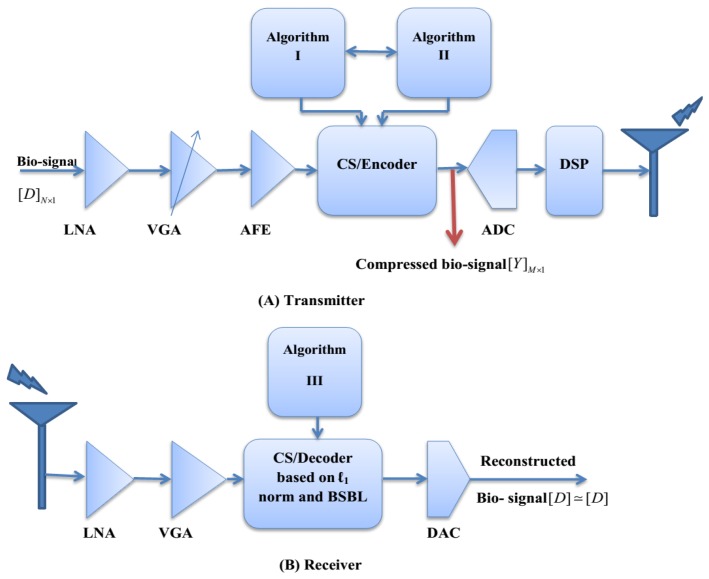
The proposed system architecture (**A**) Transmitter (**B**) Receiver.

Figure 2 illustrates the proposed block diagrams based on the analog-CS theory for the transmitter and receiver sides of an ultralow power wireless sEMG sensor. The transmitter side consists of the following parts: (1) sEMG amplifier including Low Noise Amplifier (LNA) and Variable Gain Amplifier (VGA) with variable gain in 60–80 dB for frequency range from 15 Hz to 1 kHz [26,27] (in our work, MD3880 for VGA and INA329 for LNA have been used); (2) an Analog-Front-End (AFE) (in our work, ADS1298 has been used); (3) an analog-CS step based on two proposed algorithms; (4) 16 bits ADC to digitize the analog compressed sEMG; (5) the transmitter to send the compressed bio-signals via wireless networks to the receiver (in our work the TeleMyo 2400T G2 Transmitter at a sampling rate of 1500 samples per second has been used). The suitable CR level of a raw sEMG bio-signal for diagnosis and treatment purposes is between 10 and 30, which requires a resolution of 8–12 bits [28]. (In our work, CR = 25 with 10-bits resolution has been adopted). On the other hand, to control the CR level, the sparsity level of the bio-signal can be adjusted by applying a robust dynamic thresholding approach to the input signal [29]. We use the dynamic approaches based on the following steps: (1) the DC level of the bio-signal computes a sliding average over a window of length *w* (*w* has selected between 5 and 10 in our work); (2) set the threshold level as a fraction of the peak-to-peak amplitude; (3) control sparsity level by careful selection of the sparsifying domain [30]. At the receiver side, the original bio-signal is reconstructed by applying a robust reconstruction algorithm based on a contribution of ℓ_1_-ℓ_1_-optimization and BSBL framework for diagnostic and treatment purposes.

## 4. The Proposed Algorithms

Three new algorithms for the transmitter and receiver sides are presented. The purpose of Algorithms I and II is to generate analog compressed sEMG bio-signal at the transmitter side. The main objective of Algorithm III is to recover the original sEMG bio-signal from the compressed bio-signal at the receiver side.

### 4.1. Transmitter Algorithms

**Figure 3.**
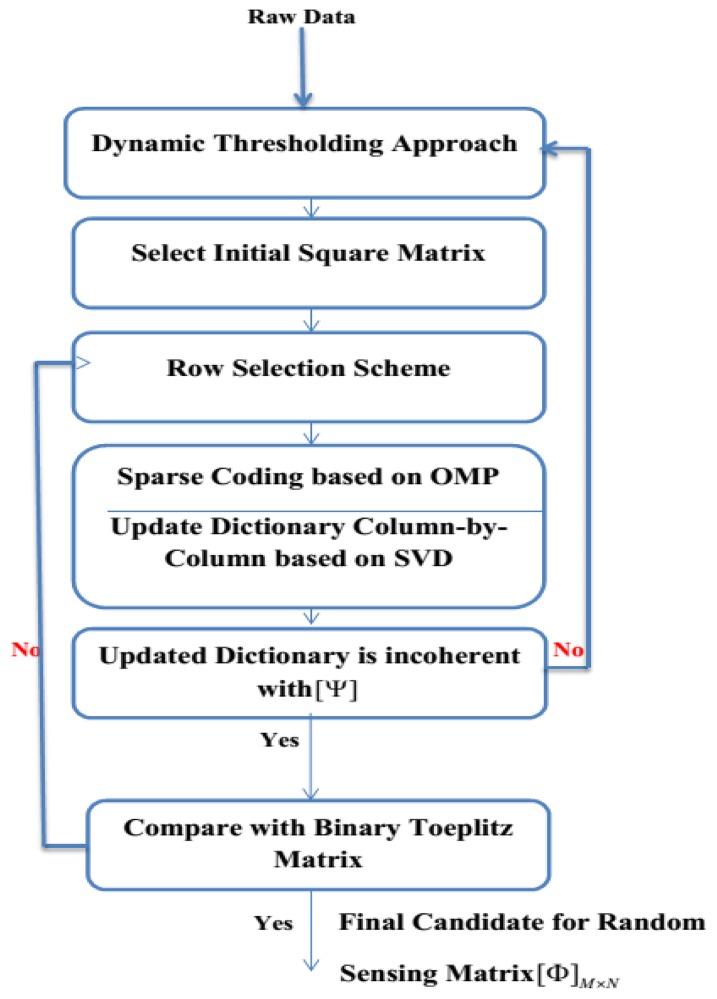
Flowchart for Algorithm II.

The fundamental purpose of Algorithm I is to generate the analog compressed sEMG bio-signal based on random sensing dictionary *ϕ* obtained from the SMS method in Algorithm II. This Algorithm consists of the following steps: (1) get the random sensing matrix *ϕ* from Algorithm II [30]; (2) examine the Restricted Isometry Property (RIP) for the selected matrix; (3) calculate the sparsity level; (4) generate the compressed bio-signal. The flowchart of Algorithm II is shown in Figure 3. The main objective of Algorithm II is to select the best candidate for the random sensing matrix *ϕ* in the CS scenario to ensure high accuracy in the reconstruction process. This algorithm comprises of the following steps. (1) Apply dynamic thresholding to the raw sEMG bio-signal; (2) Select the initial square matrix; (3) Employ the row selection scheme [31]; (4) Generate sparse coding based on Orthogonal Matching Pursuit (OMP) [32]; (5) Update the initial matrix; (6) Compare the updated matrix with the Binary Toeplitz (BT) matrix [33]; (7) Verify the incoherence degree between the updated matrix and the sparsity bases; (8) Nominate the best candidate for the random sensing matrix *ϕ* in order to generate the compressed analog bio-signal at the transmitter side of the wearable wireless sEMG sensor. Tables 2 and 3 show the pseudo-codes for Algorithms I and II, which are applied to the transmitter side of sEMG sensor.

### 4.2. Receiver Algorithm

The main objective of the robust reconstruction algorithm based on a combination of ℓ_1_-ℓ_1_-optimization and BSBL framework is to recover the original bio-signal from the compressed bio-signal at the receiver side. Based on the sparsity level of the sEMG signal, the reconstruction process at the receiver side could be selected as either ℓ_1_-optimization or ℓ_1_-ℓ_1_-optimization and BSBL framework in order to reconstruct the received bio-signal with high probability and enough accuracy [34]. The ℓ_1_-ℓ_1_-optimization is applied to the received sEMG-neuropathy signals, which are sparse in the frequency domain as [34]:

(4) $$\text{min}\left({\left\|D\right\|}_{1}+\lambda |FD{\|}_{1}\right)\;s.t.\;{\left\|C-\Phi D\right\|}_{2}≺\varepsilon$$

where *λ* is a positive constant and *F* is a *N* x *N* Fourier transform matrix. The ℓ_1_-ℓ_1_-optimization idea comes from multi-ℓ_1_-optimization, which can expressed as [34,35]:

(5) $$\text{min}(\sum_{i=1}^{N}\left(\lambda_{i}{\left\|\Psi_{i}D\right\|}_{1}\right)\;s.t.\;{\left\|C-\Phi D\right\|}_{2}≺\varepsilon$$

where *λ*_i_ is a positive constant. In the frequency domain Ψ is changed to *F*, which is an *N* x *N* Fourier transform matrix. In another scenario, if the transmitter receives sEMG-healthy or sEMG-myopathy signals that are sparse in the time domain, the ℓ_1_-optimization is applied to the received bio-signal in order to reconstruct the original bio-signal. The ℓ_1_-optimization is defined as [35]:

(6) $$\text{min}\left({\left\|D\right\|}_{1}\right)\;s.t\;{\left\|C-\Phi D\right\|}_{2}≺\varepsilon$$

Table 4 shows the pseudo-code for Algorithms III.

**Figure 4.**
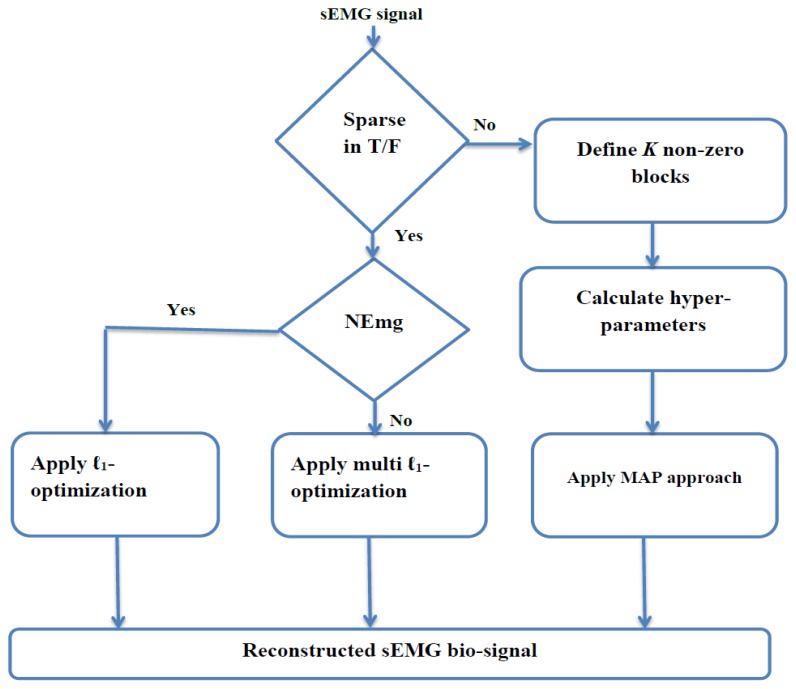
Flowchart for Algorithm III.

In the last scenario, the BSBL framework is applied to the received bio-signals that are not sparse in the time or frequency domains but are sparse in terms of non-zero blocks. This framework divides the non-sparse bio-signal into a set of non-overlapping blocks with a few non-zero blocks [36]. By employing this framework, a non-sparse signal can be partitioned into a concatenation of non-overlapping blocks of which only a few blocks are non-zero [37]. Therefore, the main structure of this framework is to explore and exploit the intra-block-correlation in terms of entry values within each block [38,39]. The flowchart of Algorithm III is shown in Figure 4. This framework consists of the following steps [40,41]. (1) Define the input bio-signal into a set of non-overlapping blocks with only non-zero blocks and mutually uncorrelated to each other; (2) Determine the Gaussian distribution property for each non-zero block with two hyperparameters that can control the block-sparsity level; (3) Estimate the posterior of the original bio-signal by applying the Maximum-A-Posteriori (MAP) approach [42–44]; (4) Update the initial original bio-signal by applying two learning rules for hyperparameters based on Auto-Regressive (AR) method [45,46].

**Figure 5.**
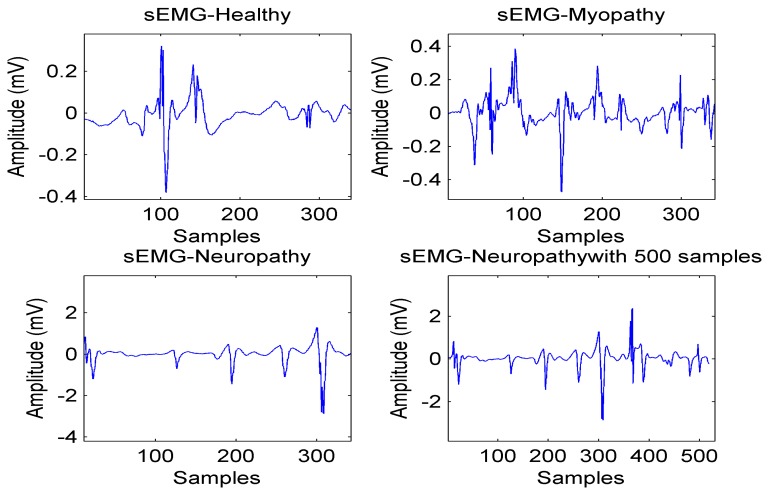
Healthy, neuropathy and myopathy sEMG signals.

As a result, the reconstruction algorithm at the receiver side consists of the following steps. (1) Check the sparsity in terms of either the number of non-zero coefficients in the time or frequency domains or the number of non-zero blocks; (2) Apply ℓ_1_-optimization for healthy and myopathy bio-signals (as they are sparse in the frequency domain) and ℓ_1_-ℓ_1_-optimization for neuropathy bio-signals; (3) Apply BSBL framework for sparsity in terms of number of non-zero blocks. Figure 5 illustrates four types of sEMG signals with the length of N = 350 to 500 and the average computing time is equal to 15.26 s.

## 5. Simulation Results

For the simulation results, several hours of sEMG bio-signals that are sparse in time or frequency domains are selected from the following databases: (1) PhysioNet (PhysioBank ATM including EMG-healthy, EMG-myopathy, and EMG-neuropathy bio-signals and annotations) [8]; (2) EMG Bank [9]; (3) EMG project lab [10].

For simulation results, the following assumptions have been made. (1) The Analog-CS approach was performed using 250 × 1024 BT matrix *ϕ*; (2) Length of window for dynamic thresholding is chosen between 5 and 10; (3) The sEMG healthy (H), neuropathy (N), and myopathy (M) signals with 1024 samples dynamically threshold at 10% to control the sparsity level; (4) CR = 25% with 10 bits of resolution. Table 5 depicts a comparison on the analog-CS and the digital-CS in terms of sensitivity (SEN) and specificity (SPE). The SEN and SPE are calculated by the following equations [35]:

(7) $$\begin{array}{ll} \text{SEN}=T_{P}/\left(T_{P}+T_{N}\right), & \text{SPE}=T_{N}/\left(T_{N}+F_{P}\right) \end{array}$$

where *T_P_* is true positive, *T_N_* true negative, *F_P_* is false positive, and *F_N_* is false negative.

**Figure 6.**
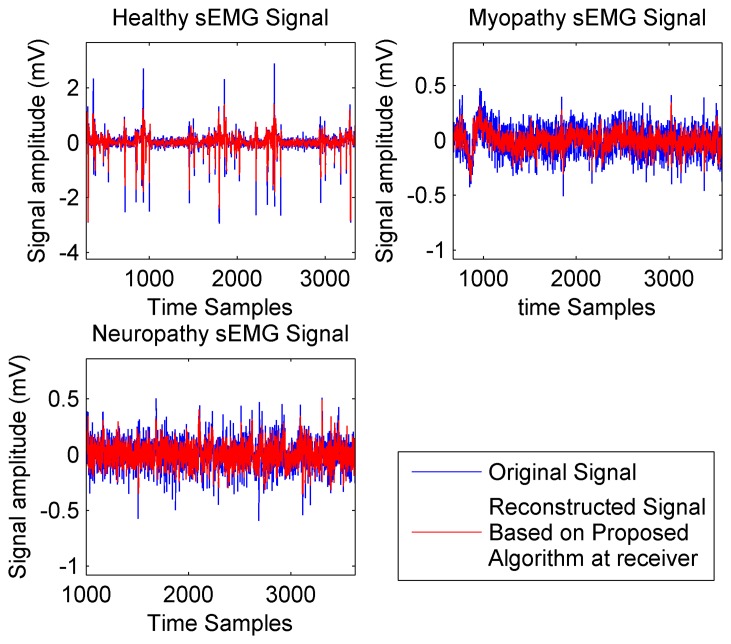
Performance of reconstruction process.

**Figure 7.**
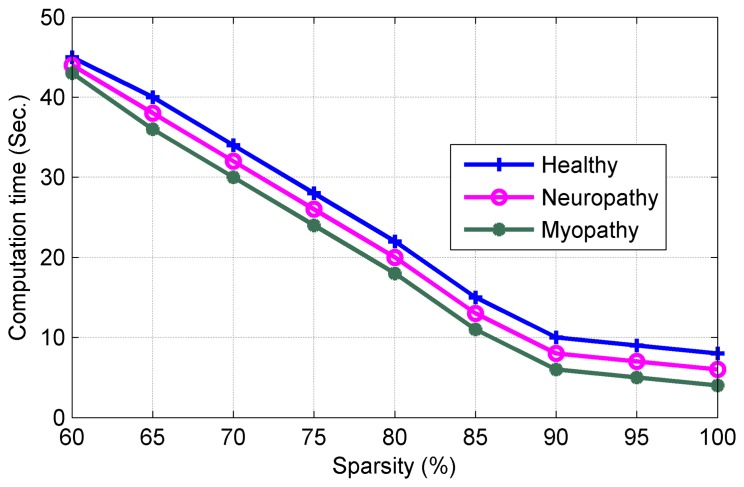
Computation time.

It should be noted that the proposed analog-CS algorithm shows very high SEN and SPE compared with current digital-CS. Figure 6 compares the quality of reconstruction process with *F_s_* = 2 kHz for three types of sEMG signals. It can be observed from Figure 6 that the proposed reconstruction process exhibits very good level of accuracy at the receiver side for recovering the original bio-signal. Figure 7 plots the Computation Time (CT), which is an important factor to minimize the power consumption *vs*. the sparsity level.

It can be noted from Figure 7 that increasing the sparsity level reduces the CT (in our work to 9.01 s at sparsity level of 98.9%).

**Figure 8.**
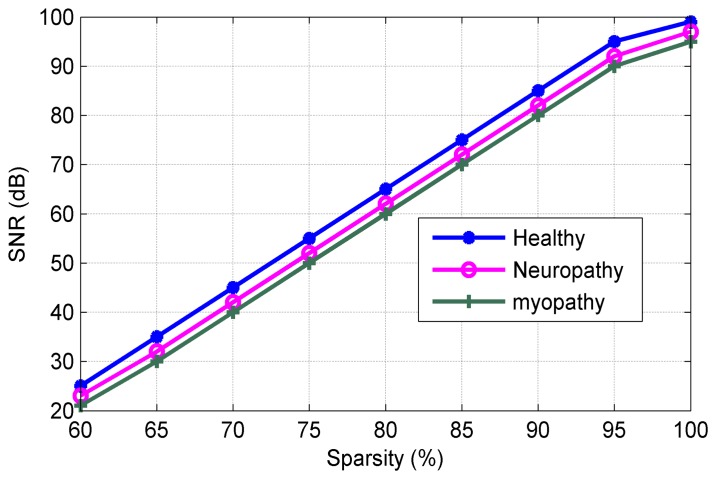
SNR for three types of sEMG signals.

**Figure 9.**
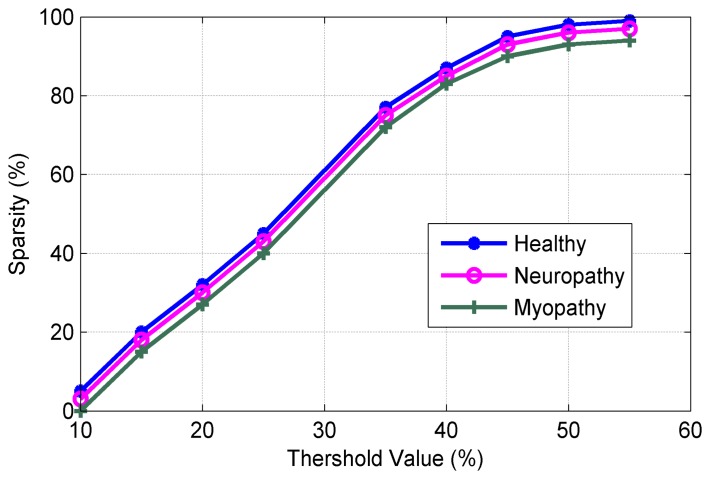
Sparsity level.

Figure 8 shows the SNR versus of sparsity level. It can be observed from Figure 8 that the proposed algorithms exhibit excellent performance in SNR (in our work to 89.2 dB at sparsity level of 98.9%). Figure 9 plots the sparsity level versus of the threshold value. As mentioned earlier, the suitable value of the thresholding process increases the sparsity level for the sEMG bio-signal, which is an important parameter in the CS scenario. It can be noted that the good level sparsity is obtained by increasing the threshold value (in our work the threshold percentage was selected at 30%).

**Figure 10.**
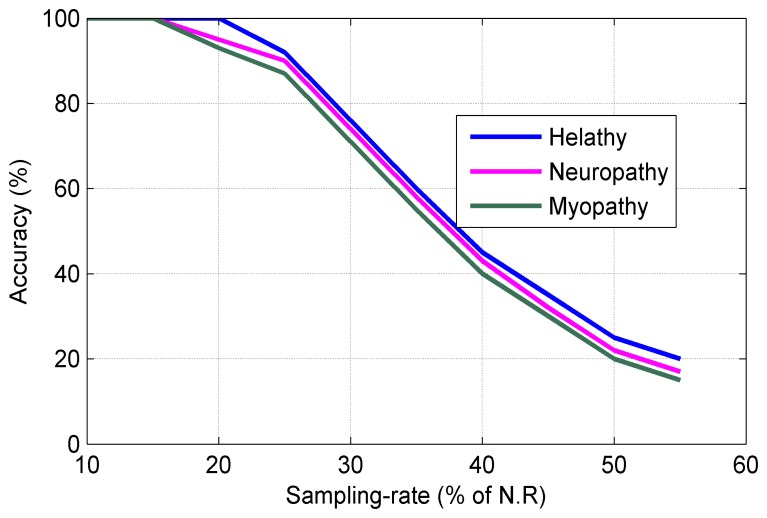
Sampling rate.

**Figure 11.**
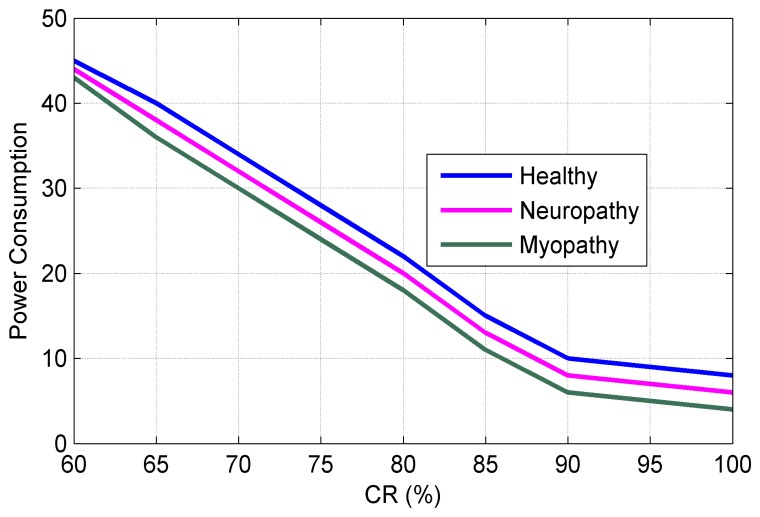
Normalized power consumption.

**Figure 12.**
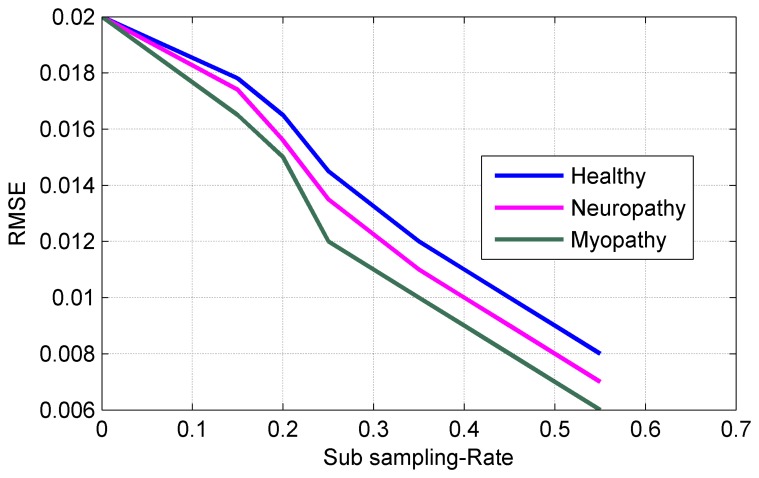
RMSE.

Figure 10 shows the sampling rate (percentage of NR) *vs*. the reconstruction accuracy for three types of sEMG bio-signals. It can be seen from Figure 10 that the sampling rate can be reduced to 25% of NR without sacrificing of the performance in the reconstruction process. Figure 11 plots the Normalized Power Consumption (NPC) vs. CR for three types of sEMG bio-signals. It can be seen from Figure 11 that power consumption can be minimized to 40% at CR = 25 in our work. Figure 12 plots the RMSE in terms of Sub-Sampling Ratio (SSR), which is 1/CR, for three types of sEMG bio-signals. The RMSE is calculated via the following formula [34]:

(8) $$\sum_{l=1}^{L}\left({\left\|D_{1}-\hat{D}\right\|}_{2}/{\left\|\hat{D}\right\|}_{2}\right)$$

where *D_l_* is the normalized original sEMG bio-signal in the *l^th^* Monte Carlo Simulation (MCS), *D̂* is the normalized estimated sEMG bio-signal in the *l^th^* MCS, and *L* is the number of Monte Carlo Simulation, which is selected to be 50 in our simulation. It can be seen from Figure 12 that all RMSE values decrease with decrease of CR or the increase of sub-sampling ratio. The MCS is a method for obtaining numerical results, which relies on many simulations by varying parameters within statistical constraints [35].

**Figure 13.**
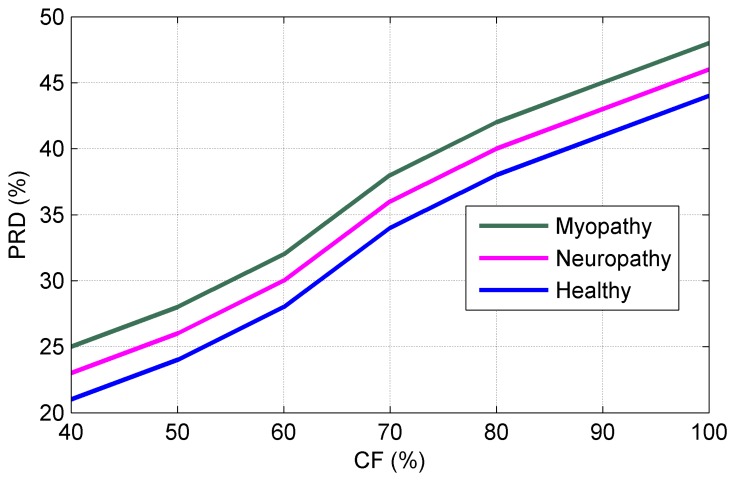
PRD.

Figure 13 shows the PRD vs. Compression Factor (CF) for three types of sEMG bio-signals sampled at 8 kHz and digitized with 4 bytes/sample. The *PRD* are defined as [35]:

(9) $$\begin{array}{l} \text{PRD}=\sqrt{(\sum_{i=1}^{N}{\left(D\left[i\right]-\hat{D}\left[i\right]\right)}^{2}/(\sum_{i=1}^{N}D^{2}\left[i\right]} \end{array}$$

where *D*[*i*] is the original sEMG bio-signal, *D̂* [*i*] is the reconstructed sEMG bio-signal, and *N* is the total number of samples. The *CF* is calculated by:

(10) $$CF=D_{b}-C_{b}/D_{b}*100$$

where *D_b_* is the number of bits for the original sEMG bio-signal and *C_b_* is the amount of bits necessary for processing the compressed sEMG bio-signal. It can be seen from Figure 13 that decreasing CF minimizes the PRD, which means good PRD level is achieved at smaller number of bits necessary for storing the original sEMG bio-signal. Highlighted in Figure 13 are the lower PRD values obtained with smaller CF. Table 6 summarizes the results on SSR and RMSE for three types of sEMG signals. Since processing long-term sEMG bio-signals involves sampling frequency from 0.5 kHz to 6 kHz and quantization with 4 byte/sample and generates huge bits of information, coding with fewer bits based on the analog-CS theory is an optimal simulation for minimizing the power consumption and sampling rate. Table 7 summarizes the results on CT in seconds and SNR in dB for three types of sEMG signals. Measurements in Table 7 illustrate values of 98.82 dB, 96.83 dB, and 94.46 dB for SNR at the sparsity level of 98.99%. Table 8 summarizes the results on NPC, Sampling-Rare Reduction (SRD), and Accuracy (A) of the reconstruction process at the receiver side for three types of sEMG signals. Measurements in Table 8 illustrate values of 0.42, 0.41, 0.40 for NPC at SRD = 25%.

## 6. Discussion

The existing long-term sEMG monitoring systems with multiple channels suffer from serious problems such as very large amount of data from sampling, limited processing capacity, limited storage, low transmission rate and power, bulky size, poor mobility, and high power consumption. Therefore, they are not suitable for wearable wireless applications that must have small size, excellent mobility, low power consumption, and high transmission rate. As most of the power in a wearable wireless sEMG sensor is dissipated for transmitting bio-data to the receiver, which may reside in a smartphone or any portable device, an optimal solution for minimizing the power consumption is to decrease the amount of bio-data and reduce the duty cycle. Therefore, real-time analog data compression plays an important role for wireless portable devices. Wearable wireless sEMG sensors based on the analog-CS theory aim to establish low power and low sampling rate algorithms for the long-term recording of the electrical activity produced by muscles, which are very useful for treatment and diagnostic purposes as well as for detection of various pathologies.

In the analog-CS scenario at the transmitter side, the input analog sEMG bio-signal [*D*]*_N ×_*_1_ is compressed to output analog bio-signal [*D*]*_M_*_× 1_ with *M ≪ N.* This work has presented a novel approach based on the analog-CS theory, which consists of three algorithms for the acquisition and reconstruction of analog data for a wearable wireless sEMG sensor. At the transmitter side, two new algorithms have presented in order to apply the analog-CS theory before ADC. At the receiver side, a reconstruction algorithm based on a combination of either ℓ_1_-ℓ_1_-optimization or ℓ_1_-optimization and BSBL framework has presented to reconstruct the original signals from the compressed signals with high probability and enough accuracy.

The proposed algorithms enable recovery of the full sEMG bio-signal while simultaneously compressing the amount of collected analog data at the transmitter side and relaxing the noise at the receiver side. The proposed architecture has offered lower energy consumption compared with the digital-CS scheme and enabled analog data compression. Tables 9, 10 and 11 demonstrate the comparisons on power consumption, accuracy, SNR, CT, and Classification Accuracy (CA) based on K-Nearest Neighbor (K-NN) [47] for three types of sEMG bio-signals for the digital-CS and analog-CS scenarios. The highlights in Tables 9, 10 and 11 can be classified as follows. (1) By applying Algorithms I and II at the receiver side of the sEMG bio-sensor, lower values for PRD and CT are obtained; (2) By employing the robust reconstruction algorithm based on a combination of multi-ℓ_1_-optimization and BSBL framework at the receiver side of the sEMG bio-sensor, lower values for SNR and PC as well as higher values for classification and reconstruction accuracy are obtained. Based on the results in Tables 9, 10 and 11, the analog-CS approach is an optimal solution for establishing low power wireless portable sEMG long-term recording systems. Table 12 presents a comparison on Power Reduction (PR) at the transmitter and receiver sides for the digital-CS approach based on ℓ_1_-optimization in terms of different values of CR. Table 13 demonstrates the PR at the transmitter and receiver sides for the analog-CS approach based on ℓ_1_-ℓ_1_-optimization and BSBL framework for three types of sEMG signals.

It can be seen from Table 13 that greater reduction of power consumption is achieved by applying analog-CS to the transmitter and receiver sides of a sEMG bio-sensor. Measurements in Table 13 show 17%, 37%, 21% and 45% power reduction at 50% NR and 7%, 12%, 9% and 15% power reduction at 25% NR for three types of sEMG bio-signals, respectively

## 7. Conclusions

Today's healthcare systems are based on wired/fixed sensors because in many cases biomedical sensors for medical monitoring are not yet wireless. This creates the need for the implementation of new wireless bio-sensors for long-term recording and monitoring of bio-signals. The benefit of using wireless healthcare systems based on wearable wireless bio-sensors can be divided into two areas.

One area is the use of new wireless technological solutions for individually based multi-parameter monitoring at home. It means patients with chronic diseases, as well as the constantly growing population of seniors, will benefit from treatment and medical monitoring at home or workplace. Moreover, unrestricted freedom of movement implies the use of wireless and even implanted biomedical sensors that greatly enhance home monitoring and follow-up of medical conditions. The second area of benefit rests in increasing the efficiency of treatments at hospital and medical centers. The ambulatory systems based on new technologies can become lighter, smaller, and capable of recording multiple signals up to 48 h. The recorded signals are saved in flash-type memories, which can be transferred to the hospital and medical centers for further actions. On the other hand, the increase of heath costs urgently prompts the development of ambulatory systems to reduce the number of patients going to hospital and medical centers.

Therefore, it is necessary to design portable, low power, low sampling rate, high performance wireless health care systems that can deliver healthcare services anywhere at any time. This paper has presented an analog-based CS theory procedure that consists of three novel algorithms to design and implement the wearable wireless sEMG bio-sensor architecture for sEMG bio-signals, which are sparse in both the time and the frequency domains. At the transmitter side, two new algorithms have been presented in order to apply the analog-CS theory before ADC to maximize the processing speed by minimizing both the load of processing time and the duty cycle. At the receiver side, a faithful reconstruction algorithm based on the combination of multi-ℓ_1_-optimization and BSBL framework has been presented to reconstruct the original bio-signals from the compressed bio-signals with high accuracy. In addition, this work has presented an analog-based CS theory procedure that consists of two interesting properties: (1) reduction in power consumption, which significantly lowers the average bio-sensor power; (2) a reconstruction process robust to noise, because the noise is not sparse on the sparsity basis. Therefore, the resolution of the data acquisition step, and hence the ADC, can be relaxed without undermining the quality of the reconstruction. The proposed architecture has reduced the sampling rate down to 25% of NR, power consumption to 40%, PRD to 24%, RMSE to 2%,and total computation time from 22 s to 9.01 s, which provide good background for establishing wearable wireless healthcare systems. In addition, the proposed architecture simultaneously has recovered the original bio-signals with good level of accuracy and SNR greater than 95.8 dB. Wireless sEMG systems based on the proposed architecture and the three novel proposed algorithms will be able to improve healthcare services for patients not only in hospital or at medical center but also at their homes and workplaces, to improve the overall quality of life and to minimize healthcare costs.

## 8. Future Works

We intend to develop the tools for wireless sEMG bio-sensor and provide them to the web-based portals. The developed tools can be designed to access the valuable data stored on medical servers. Furthermore, we will develop a hybrid reconstruction algorithm based on multi-ℓ_1_-optimization and BSBL framework for other types of multi-sparse bio-signals to decrease the computational complexity at the receiver side.

## Figures and Tables

**Table 1.** Compression techniques.

**Technique**	**Sampling Rate**	**Distortion**	**CR**
Existing methods	2*f_s_*	Medium	Medium
CS method	*f_s_*/*CR*	Low	High

**Table 2.** Algorithm I: compressed sEMG bio-signal.

**Input:** sEMG bio-signal [*D*]*_N_*_× 1_,	**Output:** Compressed bio-signla [*C*]*_M_*_× 1_
Sparsity matrix [Ψ ] *_N_*_×_ *_N_*,	
Random matrix [Φ ]*_M_*_×_ *_N_*,	
Threshold Value (TV)	

Step	Process
1.	**If** [Ψ] has RIP Property
2.	**else**
3.	*P* = 0.01
4.	**While** [Ψ] not RIP Property
5.	*P* = *P* + 0.2
6.	**end While**
7.	Calculate Sparsity level (*SP*) = (*N/N* − *K*)
8.	If *SP* ≥ 0.98, *M* ≫ *βKlog*(*N/M*)
9.	**else**
10.	**While** *SP* ≺ 0.98
11.	*TV* = *TV* + 0.01
12.	**end While**
13.	**end**
14.	Generate compressed bio-signal [*C*]*_m_*_× 1_

**Table 3.** Algorithm II: sensing matrix [Ψ].

**Input:** Raw sEMG bio-signal [*D*]*_N_*_× 1_	**Output:** Random sensing matrix [Ψ]
Sparsity matrix [Ψ]*_N_*_×_ *_N_*	

Step	Process
1.	Apply dynamic thresholding approach
2.	Select initial square matrix
3.	Apply row selection scheme
4.	Determine sparse coding based on OMP
5.	Update selected dictionary based on SVD
6.	Compare with Binary Topeltiz (BT) matrix
7.	Verify the incoherence degree with [Ψ]
8.	Nominate the selected dictionary as [Ψ]

**Table 4.** Algorithm III: robust reconstruction process.

**Input:** Raw sEMG bio-signal [*D*]*_N_*_× 1_,	**Output:** Reconstructed sEMG bio-signal
Sparsity matrix [Ψ]*_N_*_×_ *_N_*,	
Random matrix [Φ[*_M_*_×_ *_N_*	

Step	Process
1.	If [*D*] is sparse in time or frequency
2.	If [*D*] is sEMG-Neuropathy signal
3.	*P* = 0.01
4.	Apply ℓ_1_-optimization by *min* ‖*D*‖_1_ *s.t C* = Φ*D*
5.	Determine reconstructed bio-signal **endif**
6.	**elseif** [*D*] is sEMG-Healthy or sEMG-Myopathy
7.	Apply ℓ_1_-ℓ_1_ -optimization
8.	If *SP* ≥ 0.98, *M* ≫ *βKlog*(*N*/*M*)
9.	Determine reconstructed bio-signal **endif**
10.	**elseif** [*D*] is sparse in tems of *K* non-zero blocks
11.	Calculate hyper parameters (*γ*, *β*)
12.	Obtain Σ_0_ = *diag*(*γ, β*)
13.	Initial estimation of [*D*] ≃ *N*(*0*, Σ_0_)
14.	Apply MAP approach
15.	Determine reconstructed bio-signal **endif**
14.	**end**

**Table 5.** Comparison of analog-CS and digital-CS.

**Algorithm**	**SEN. %**	**SPE. %**
Proposed analog-CS (Transmitter)	99.9	98.2
Current digital-CS (Transmitter)	87.2	86.2
Proposed analog-CS (Receiver)	99.2	97.2
Current digital-CS (Receiver)	84.2	85.6

**Table 6.** Comparison on RMSE.

**SSR**	**RMSE(H)**	**RMSE(N)**	**RMSE(M)**
0	0.0200	0.0200	0.0200
0.2	0.0168	0.0144	0.0125
0.4	0.0144	0.0120	0.0110
0.6	0.0130	0.0110	0.0105
0.8	0.0120	0.0102	0.0100
1	0.0108	0.0102	0.0102
1.2	0.0108	0.0102	0.0102

**Table 7.** Comparison on SNR and CT.

**Sparsity %**	**SNR(H)**	**CT(H)**	**SNR(N)**	**CT(N)**	**SNR(M)**	**CT(M)**
60	8	45	4	44	2	43
65	36	45	32	37	30	36
70	46	35	42	34	40	33
75	55	28	51	27	49	26
80	65	22	61	21	58	20
85	76	12	72	11	69	10
90	87	8	82	7	80	6
95	96	7	92	6	90	5
100	99	6	97	5	95	4

**Table 8.** Comparison on accuracy and NPC.

**SRD%**	**A %(H)**	**A %(N)**	**A %(M)**	**NPC(H)**	**NPC(N)**	**NPC(M)**
10	100	100	100	1	1	1
15	100	100	100	0.90	0.85	0.84
20	100	95	94	0.70	0.65	0.64
25	92	90	88	0.42	0.41	0.40
30	78	74	70	0.30	0.28	0.25
35	60	58	55	0.18	0.16	0.15
40	45	44	40	0.17	0.15	0.14
45	35	31	30	0.16	0.15	0.14
50	25	21	20	0.15	0.14	0.13

**Table 9.** Comparison on healthy sEMG signals.

**Algorithm**	**PRD%**	**SNR(dB)**	**NPC**	**CT (s)**	**CA%**	**Reconstruction Accuracy%**
Digital-CS	0.0350	87.3	0.820	18.02	93.24	95.52
Analog-CS	0.0108	98.9	0.420	6.02	98.23	99.98
Existing Approach	0.056	82.1	0.93	28.30	91.85	87.24

**Table 10.** Comparison on myopathy sEMG signals.

**Algorithm**	**PRD%**	**SNR (dB)**	**NPC**	**CT (s)**	**CA%**	**Reconstruction Accuracy %**
Digital-CS	0.0341	85.2	0.815	19.25	92.85	94.92
Analog-CS	0.0102	96.7	0.415	5.24	98.89	99.97
Existing Approach	0.053	80.15	0.91	29.90	91.23	89.34

**Table 11.** Comparison on neuropathy sEMG signals.

**Algorithm**	**PRD %**	**SNR (dB)**	**NPC**	**CT (s)**	**CA %**	**Reconstruction Accuracy %**
Digital-CS	0.0362	84.1	0.831	20.22	93.99	94.83
Analog-CS	0.0100	95.9	0.400	4.27	99.01	99.12
Existing Approach	0.067	79.8	0.94	29.90	91.11	86.99

**Table 12.** Comparison on Power Reduction based on digital-CS.

**CR%**	**T. PR (H) %**	**T. PR (H) (N) %**	**T. PR (M) %**	**R. PR (H) %**	**R. PR (N)%**	**R. PR (M) %**
55	7	12	13	21	25	26
50	6	10	12	20	24	25
45	5	9	10	18	21	24
40	3	7	8	10	11	12
35	2	6	7	8	9	10
30	1.5	5.25	6	7	8	9
25	1	4	5	6	7	8
20	0.5	3	4	5	6	7

**Table 13.** Comparison on power reduction based on analog-CS.

**CR%**	**T. PR (H) %**	**T. PR (H) (N) %**	**T. PR (M) %**	**R. PR (H) %**	**R. PR (N) %**	**R. PR (M) %**
55	17	21	21	37	45	45
50	15	20	20	35	41	43
45	10	18	19	34	40	41
40	9	10	15	16	18	22
35	8	7	13	10	15	18
30	7	8	12	9	12	15
25	6	7	10	8	10	12
20	5	6	8	7	9	10
